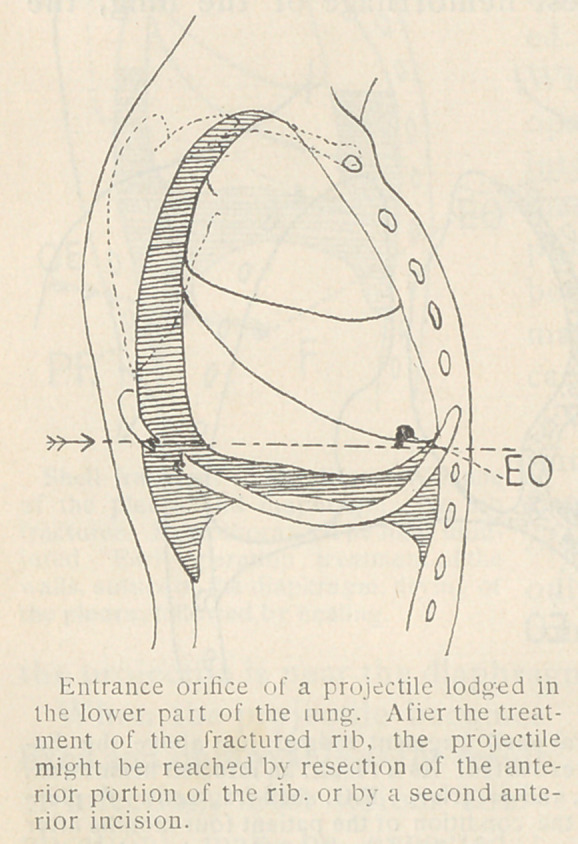# Wounds of the Pleura and of the Lung by Projectiles

**Published:** 1918-05

**Authors:** 


### SURGICAL

**Wounds of the Pleura and of the Lung by Projectiles.** By J. L. Roux-Berger, Médecin-Major de 2e Classe. Trans. and Abs. from the *Lyon Chirurgical*, January-February, 1918.

The author concludes, from experiences with infection of chest wounds, that all such wounds should be operated, if only for the purpose of treating the walls of the chest, which may be an important source of infection.

It has often been said that a surgeon should operate a chest in three cases : 1) a widely exposed pleura, 2) hemorrhage, 3) infection.

Such a conception is too limited. Surgery should first be used as a means of prevention. Every chest wound requires operation as early and as complete as possible. Such operation applies to the wall, the pleura, the lung, and to the extraction of projectiles. Only in this way can infection and hemorrhage surely be prevented.

The following exceptions, however, may be made :

In a case with multiple wounds, the chest wound need not be operated unless there are severe complications.

Wounds with a strictly punctiform orifice need not be operated so long as there are no signs of hemorrhage, and the projectile tract does not require verification.

The author considers the following instruments indispensable :

Two intercostal retractors (Tuffier) — the small model with 3 cm. valves and the large model with 6 cm. valves.

Heart-shaped forceps (pliant for grasping the lung)

Intestinal forceps (Chaput), which are excellent for the lung because they do not require pulling.

Mayo's needles for suture.

Frontal mirror.

Complete X-ray installation.

*Treatment of the Walls.* So far as the soft parts are concerned, the treatment of the walls is the same as in all war surgery, the technique for which is now in a highly developed stage, and consequently is unattended by serious obstacles.

Treatment of the bone, however, is more difficult, and requires the very greatest care, especially when resection for badly shattered ribs is practised. The smallest and most insignificant fracture of a rib is always important enough to require resection.

Since any parietal lesion may cause pleural infection, one ought never to overlook the seat of a costal fracture, however difficult the operation or however serious the condition of the patient.

In the case of a patient who had a shrapnel wound in the right clavicular region, the X-ray examination showed the projectile to be lodged near the first dorsal vertebra. The first rib, which was fractured, was resected, and a portion of the thorax was removed so as to permit of a lung suture and the drying of the pleura. On account of the weakness of the patient, only a few bone splinters were removed and the operation stopped. The next day a hemothorax had again appeared with signs of serious infection. The neck of the first rib was resected from behind and the shrapnel removed, but it was too late to save the patient who died the next day from acute pleural infection.

A surgeon should never hesitate to sacrifice the whole or a part of the clavicle by a sub-periostal resection. It affords better lighting and permits of the examination of the large vessels at the base of the neck.

The treatment of the ribs in the sub-scapular region is still more difficult. They are deeply set, the muscles are thick, and the movement of the scapula is very limited. Under no conditions should these ribs be completely isolated.

When the scapula is fractured, a very wide resection must be performed, for in fractures of this nature, fistulae may be formed all along the bone, causing very serious infection. By this procedure, also, it is easier to reach the rib.

Even when the scapula itself is not] fractured, the surgeon must reach the rib through the scapula by wide resection unless the fracture of the rib is near the edge of the scapula.

Shell fragment in the liver. Wounds of the pleura and diaphragm. No rib fractures. Hemothorax. The lung uninjured. Early operation: treatment of the walls, suture of the diaphragm, drying of the pleura, followed by healing.

*Treatment of the Pleura and of the Lung.*

a) *Projectile in the pleura.* Every intra-pleural projectile must be removed. When the projectile is free, infection is much to be feared. The treatment is very simple. When the thorax has been laid open, the whole hand can be thrust into the pleural cavity, and in this manner every corner may be explored. After the projectile has been located and removed, the lung may be examined and the pleura carefully dried and closed.

When the projectile is fixed and immovable from the start, there is probably no lesion of the lung or of the layers of the pleura. The only important clinical sign is the contraction of the abdomen when the projectile is near the diaphragm or peritoneum.

When the projectile becomes fixed and immovable as the result of infection, most serious complications may be expected: infected hemothorax, retraction of the lung, massive infection of the pleura, and acute pleuro-pulmonary complications.

In one instance, a man had a shell fragment at the base of the right thorax. The projectile was not removed. A hemothorax occurred and was drained. An intra-thoracic abscess was opened. Four months later the pleura was fistulous and an X-ray examination revealed a greatly thickened wall of the pleural cavity drained at the bottom. There was no liquid, and on the bottom of this pocket against the

Piercing wound of the base of the thorax by ball. Wound of the liver and the diaphragm. Tearing of the edge of the lung. Hemothorax. Rib fractures. Suture of the wounds and of the diaphragm. The walls treated. Healing.

diaphragm was a very large shell fragment. If a complete operation for the removal of the projectile had been performed at an early stage, with removal of the projectile, this infectious complication would have been avoided.

b) *Treatment of the Hemothorax.* Preventive measures are the most important. The slightest hemorrhage of the lung, the diaphragm, or the wall, should be sutured immediately, and thus hemothorax with its infectious possibilities may in most cases be avoided. The perfect drying of the pleural cavity and the suppression by suture or ligature of all sources of secondary exudation, however slight, are important means of preventing post-operative complications. In this respect all the care bestowed in military surgery upon wounds of the soft parts should be exercised in wounds of the cavity.

An intra-pleural shell fragment lodged just above the diaphragm and not extracted. As a result, an infected hemothrax, a pleural pocket, and pleurisy. E.O, shell fragment. P. pleurisy. This was the condition of the patient four months after the superficial treatment of the wall. This serious infection could have been prevented by the early extraction of the projectile.

c) *Drainage of the Pleura.* If the case is treated within eight or ten hours, it is generally useless to drain the pleura. If, however, there are any large fragments of shell or clothing, it must be drained, with every possible precaution against the entrance of air. Dakin's solution may be used if necessary.

d) *Intra-pulmonary Projectiles.* Any projectile, even if small and far removed from the pleura, may lead sooner or later to hemorrhagic or infectious complications. Hence any such fragments ought to be removed at the start.

If the projectile has not penetrated very far into the lung, it is easy to reach it through the entrance orifice. If, however, it is deeply imbedded, and especially if the fragment is small, the procedure is much more difficult. It is then frequently impossible to reach it through the entrance orifice. An incision must therefore be made as near as possible to the projectile, so as to facilitate its removal. For such purposes a complete X-ray installation is indispensable.

All lung wounds, however insignificant, require operation and treatment, for they are a source of danger to the pleura. Such operation may consist in clearing up the orifice, excising the edges, and sponging out the tract.

Entrance orifice of a projectile lodged in the lower part of the lung. After the treatment of the fractured rib, the projectile might be reached by resection of the anterior portion of the rib, or by a second anterior incision.

Sometimes a more elaborate operation is indicated, such as is often necessary in the treatment of the soft parts.

e) *Post-operative Condition.* The author states that he has never observed shock in any case of chest wound. Serious consequences are due either to excessive hemorrhage or to the bad condition of the lung. All such complications indicate immediate intervention. No post-operative shock has been observed. The earlier and the more complete the operation, the less opportunity there is for hemorrhage and infection.

The article is profusely illustrated and accompanied by careful statistics and complete details of a considerable number of cases. We are indebted to the *Lyon Chirurgical* for permission to reproduce the plates which accompany this abstract.

## Figures and Tables

**Figure f1:**
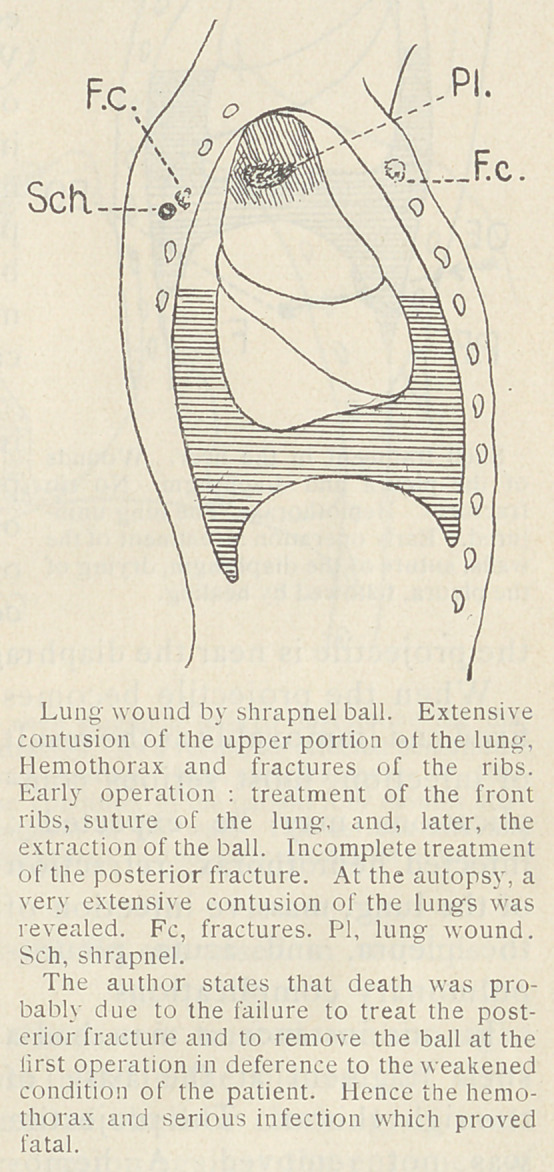


**Figure f2:**
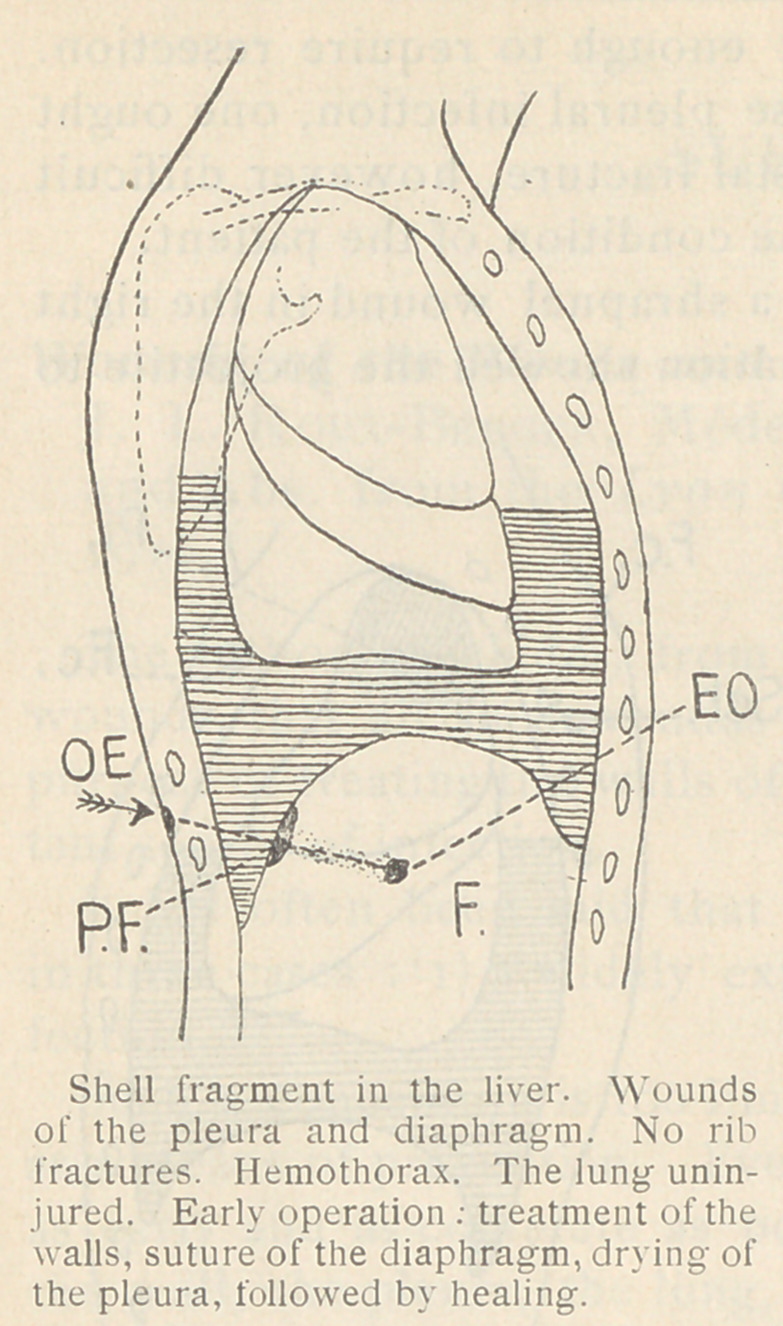


**Figure f3:**
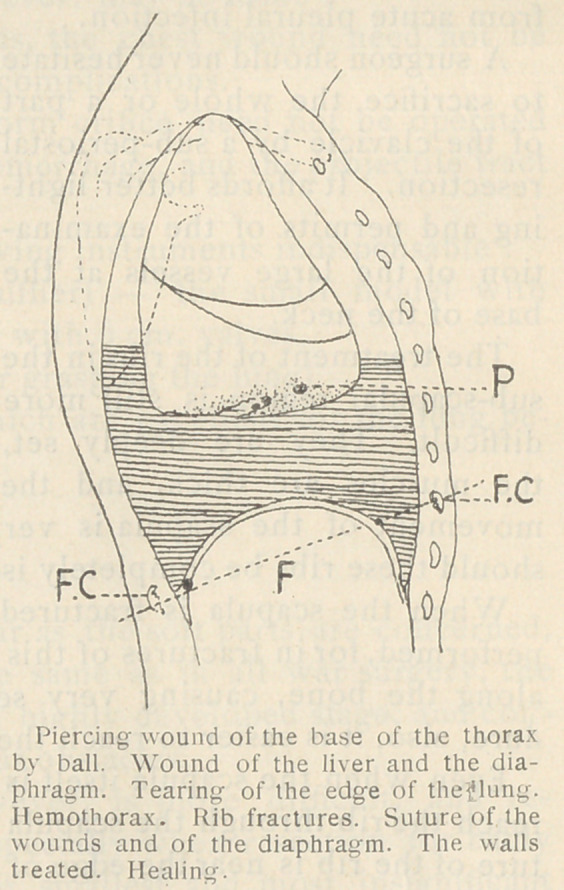


**Figure f4:**
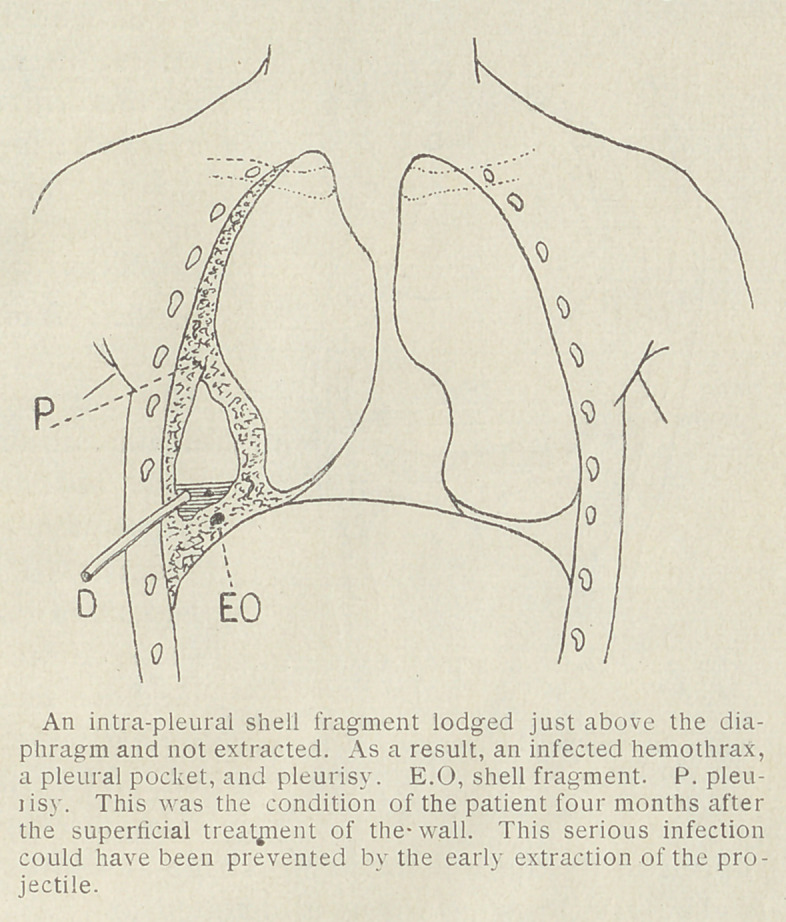


**Figure f5:**